# Comprehensive management of hemodialysis catheter-related bloodstream infections: a narrative review

**DOI:** 10.3389/fcimb.2026.1822898

**Published:** 2026-06-02

**Authors:** Yiran Ge, Yuqing Wang, Xiaojie He, Yaqing Wang, Xiaodong Li

**Affiliations:** 1 Graduate School of Hebei Medical University, Shijiazhuang, Hebei, China; 2 Graduate School of Chengde Medical University, Chengde, Hebei, China; 3 Department of Nephrology, Baoding No. 1 Central Hospital of Hebei Medical University, Baoding, Hebei, China

**Keywords:** biofilms, catheter‐related bloodstream infections, central venous catheter, drug tolerance, intelligent systems, renal dialysis

## Abstract

Tunneled central venous catheters (CVC) are essential vascular access for many maintenance hemodialysis(HD) patients, yet catheter-related bloodstream infections (CRBSI) remain a persistent complication driven by biofilm formation and multidrug-resistant organisms, creating a clinical dilemma between source control (catheter removal) and vascular access preservation. This narrative review synthesizes current evidence across HD-CRBSI epidemiology, pathogenesis, prevention, treatment, and future directions, and provides an evidence-based, decision-oriented management framework. Key contributions include: systematic distinction between extraluminal (skin → cuff → bloodstream) and intraluminal (hub contamination) routes of infection; an HD-specific pathogen profile establishing Gram-positive predominance (approximately 64% of cases, with coagulase-negative staphylococci accounting for approximately 32%); a structured clinical classification differentiating uncomplicated versus complicated CRBSI and exit-site, tunnel, and systemic infections; explicit criteria for catheter salvage (hemodynamically stable patients with uncomplicated CRBSI caused by low-virulence pathogens, mandatory clinical and microbiological reassessment at 48–72 hours, and documented clearance of bacteremia) versus mandatory catheter removal (Staphylococcus aureus, Pseudomonas aeruginosa, Candida spp., tunnel infection, septic shock, or persistent bacteremia >72 hours); systematic integration of antimicrobial lock therapy into the salvage pathway, supported by HD-specific evidence including the LOCK-IT-100 trial; prioritization of standard-of-care prevention practices (mandatory catheter hub disinfection, standardized aseptic connection/disconnection protocols, staff training with care bundles) over experimental strategies; and identification of key research priorities including standardized CRBSI surveillance methodologies, prospective validation of AI-assisted diagnostic tools, and rigorous evaluation of next-generation preventive technologies.

## 1 Introduction

Hemodialysis (HD) serves as a vital life-sustaining treatment for patients with end-stage renal disease (ESRD), whereas secure and effective vascular access constitutes the cornerstone for successful HD implementation (Flythe and Watnick, 2024).

Among various vascular access modalities, central venous catheters (CVC) remain indispensable in clinical practice. Data indicate that in countries such as Australia, the United States, and Canada, CVC are employed in approximately 80% of incident HD patients and 17–45% of prevalent cases (Opoku-Asare et al., 2023). The 2023 report from the United States Renal Data System underscores that, compared to alternative accesses like arteriovenous fistulae, CVC use is associated with a 2–3-fold increase in bloodstream infection risk, hospitalization rates, and mortality (Afzal and Silberzweig, 2024). This disproportionate reliance on CVC, driven by urgent dialysis needs and limited vascular access options, directly sets the stage for the substantial CRBSI burden in this population. This review, therefore, focuses specifically on chronic hemodialysis patients with tunneled CVC.

Catheter-Related Bloodstream Infection (CRBSI) —one of the most severe complications in HD patients—significantly exacerbates morbidity, mortality, and healthcare expenditures (Lazarus et al., 2023). Current estimates suggest that roughly 10% of patients undergoing catheter-dependent dialysis experience at least one CRBSI episode annually, highlighting its substantial clinical burden (Kotwal et al., 2022).

In recent years, the emergence and dissemination of multidrug-resistant organisms (MDRO) have intensified challenges in preventing and managing Hemodialysis Catheter-Related Bloodstream Infection (HD-CRBSI). Despite numerous proposed and implemented preventive strategies globally, persistently high CRBSI rates signal an urgent need for refining and integrating existing interventions. A central dilemma in this context is the decision between catheter salvage and removal: while source control demands removal, the exhaustion of vascular access sites and the imperative of uninterrupted dialysis frequently necessitate catheter retention, creating a persistent therapeutic tension that demands a nuanced, evidence-based approach. Consequently, a comprehensive synthesis of CRBSI epidemiology, microbiological profiles, pathogenic mechanisms, and evidence-based countermeasures holds critical implications for optimizing clinical practice and patient outcomes.

## 2 Epidemiology and disease burden of HD-CRBSI

Accurately defining the infection is crucial for surveillance and management. In this review, “Catheter-Related Bloodstream Infection (CRBSI)” refers to a clinical diagnosis requiring specific microbiological evidence (e.g., differential time to positivity) confirming the catheter as the source of the bloodstream infection. This is distinguished from the broader surveillance term “Central Line-Associated Bloodstream Infection (CLABSI),” which denotes a primary bloodstream infection in a patient with a central line, without requiring definitive proof of the catheter as the source. Our discussion and the epidemiological data presented herein focus primarily on CRBSI in the hemodialysis population.

Hemodialysis catheters are broadly classified into two categories: TCC and non-tunneled catheters (NTC). TCC possess a subcutaneous Dacron cuff that serves as a mechanical barrier against bacterial migration along the catheter tract, making them the standard choice for chronic maintenance HD. NTC, which lack this cuff, are primarily intended for short-term use and are associated with a higher infection risk. Unless otherwise specified, the epidemiological data reviewed in this section pertain to chronic maintenance HD patients with TCC.

Catheter-related infections in HD patients encompass three distinct clinical entities: exit-site infections, characterized by local erythema, tenderness, or purulent discharge within 2 cm of the catheter exit site, typically without systemic involvement; tunnel infections, involving inflammation along the subcutaneous catheter tunnel beyond 2 cm from the exit site, often accompanied by systemic signs; and CRBSI, defined by positive blood cultures with the catheter identified as the source, usually presenting with fever, chills, or hemodynamic instability. This review focuses primarily on CRBSI, although exit-site and TCC are addressed as they represent important precursors and risk factors for bloodstream infection.

CRBSI exhibits distinct epidemiological characteristics worldwide, imposing a substantial disease burden. This review focuses on catheter-related bloodstream infections in patients receiving maintenance hemodialysis, with specific consideration of non-tunneled and tunneled cuffed central venous catheters. ​Large-scale surveillance data indicate significant regional and institutional variations in CRBSI incidence among HD patients, ranging from 1.1 to 5.5 cases per 1000 catheter-days (Lazarus et al., 2023). This wide range likely stems from disparities in healthcare resources, the rigor of infection surveillance standards, and variations in catheter insertion and maintenance protocols across regions. An Indian multicenter study spanning seven years analyzed laboratory-confirmed CRBSI cases across various inpatient settings and reported an overall infection rate of 8.83 per 1000 catheter-days. The study identified significant variations across clinical settings, with neonatal ICU (Intensive Care Unit) exhibiting the highest incidence (13.86 per 1000 catheter-days), followed by adult ICU and pediatric ICU. It is important to note that while this and similar surveillance data from intensive care settings provide valuable context for the general challenge of CRBSI, the epidemiological profile, risk factors, and management focus for chronic maintenance hemodialysis patients—the primary subject of this review—are distinct (Parveen et al., 2025). Data specific to the HD population, such as from Malaysia, demonstrate that patients using semi-permanent HD catheters face a higher CRBSI risk compared to AVF users, with all-cause mortality reaching 12-25% (Shahar et al., 2021). These findings confirm CRBSI as a prevalent and clinically significant complication across healthcare settings, while highlighting the need for population-specific analysis.

Beyond Asia, developed nations similarly document heavy CRBSI burdens. It should be noted that epidemiological metrics vary across studies. The incidence is most commonly and comparably reported as cases per 1000 catheter-days. However, some population-level studies employ alternative metrics to capture different aspects of the burden. For instance, an Australian nationwide prospective cohort study of 3,943 chronic kidney failure patients reported catheter-related infection rates of 24.5 per 100 patient-years, which provides crucial longitudinal and healthcare utilization insights, particularly as community-onset HD-CRBSI accounted for 8.2% of infection-related hospitalizations (Lazarus et al., 2025). The use of inconsistent metrics, however, complicates direct comparisons between studies and healthcare systems, underscoring the need for standardized surveillance and reporting. Infection risk showed strong age dependence, being significantly higher in patients under 70. Furthermore, indigenous populations including Aboriginal and Torres Strait Islander peoples exhibited heightened susceptibility, reflecting underlying health inequities influencing infection distribution.

A comparative interpretation of this epidemiological data must acknowledge the variation in reporting metrics. The burden of CRBSI is not only universal but also its distribution is profoundly shaped by healthcare system levels, patient demographics, and societal health equity. The use of inconsistent metrics, however, complicates direct comparisons between studies and healthcare systems, underscoring the need for standardized surveillance and reporting, as highlighted in discussions on improving data quality for infection prevention. The high incidence rates in developing countries are often associated with limited infection control resources, while data from developed countries highlight the challenges faced by specific high-risk populations (e.g., younger patients, indigenous peoples). This underscores the need for prevention strategies that are both universal and targeted.

The disease burden extends beyond incidence to profoundly impact clinical outcomes and healthcare utilization. CRBSI may precipitate sepsis and serious complications like endocarditis and osteomyelitis (Johansen et al., 2022). Catheter-associated thrombosis poses another serious clinical complication, demonstrating a multifactorial etiology involving intrinsic patient characteristics—particularly prior thrombotic events, hematologic disorders, and underlying malignancies—combined with extrinsic treatment-related influences including prolonged catheter dwell time and parenteral therapy requirements (Tian et al., 2021). Infections prolong hospital stays, increase costs, and raise mortality rates. Notably, 15.7% of infection-related admissions required ICU care, exacerbating healthcare system strain (Oladapo-Shittu et al., 2024). These findings establish CRBSI as a systemic threat characterized by high prevalence, complication risks, and resource consumption among HD populations, underscoring the critical need for enhanced clinical and public health interventions.

## 3 Etiology and antimicrobial resistance patterns of HD-CRBSI

HD-CRBSI demonstrates a pathogen profile in which Gram-positive organisms predominate. Staphylococcus species—particularly coagulase-negative staphylococci (CoNS) and Staphylococcus aureus—account for the majority of infections in patients with tunneled hemodialysis catheters. Surveillance data indicate that Gram-positive cocci are responsible for approximately 64% of HD-CRBSI cases, with CoNS alone implicated in roughly 32% of infections. Gram-positive infections are particularly associated with catheter colonization and biofilm formation on the luminal surface, with S. aureus bacteremia carrying a high risk of metastatic complications including endocarditis and osteomyelitis.

Gram-negative bacteria account for approximately 36% of HD-CRBSI cases, with Pseudomonas aeruginosa and Enterobacterales being the most clinically significant. Fungal pathogens, predominantly Candida species, comprise a smaller but clinically important minority, particularly in patients with prior antibiotic exposure or femoral catheterization.

In recent years, the emergence and dissemination of multidrug-resistant organisms (MDROs) have further complicated the microbiological landscape. These organisms exhibit alarming resistance patterns, particularly concerning carbapenem antibiotics, posing substantial therapeutic challenges. A multicenter Indian study of 10,042 clinical isolates reported Klebsiella pneumoniae and Acinetobacter baumannii as the most prevalent resistant Gram-negative organisms.6These resistance patterns, driven by multiple carbapenemase genes and efflux mechanisms, severely constrain available treatment options.

Beyond drug-resistant bacteria, increasing attention has been paid to CRBSI caused by opportunistic pathogens and rare microorganisms. A comparative study between Acinetobacter seifertii and Acinetobacter nosocomialis revealed significant differences in antimicrobial susceptibility patterns and carbapenem resistance mechanisms (Lee et al., 2025). Notably, A. seifertii exhibited markedly lower sensitivity to polymyxin, amikacin, gentamicin, ceftazidime, and cefepime compared to A. nosocomialis, underscoring the necessity for pathogen-specific adjustments when formulating antimicrobial treatment regimens.

Fungal infections, particularly Candida-associated CRBSI, are a serious concern in hemodialysis. In the HD population, key risk factors for candidemia include prolonged broad-spectrum antibiotic use, the presence of a central venous catheter (especially femoral site), and compromised host defenses often seen in patients with diabetes mellitus or malnutrition (Cabrera-Guerrero et al., 2025).

Biofilm formation represents another critical pathogenic feature of CRBSI-associated pathogens, serving dual functions: shielding bacteria from antibiotic attacks and host immune defenses, while facilitating horizontal transfer of antibiotic resistance genes. Current research demonstrates that polymicrobial biofilms exhibit enhanced microbial tolerance compared to monospecies communities, substantially complicating therapeutic interventions (Kurakado et al., 2025). For instance, in dual-species biofilms with Candida albicans, carbapenem-resistant Klebsiella pneumoniae (CRKP) displays elevated drug tolerance. Notably, combination therapy employing amphotericin B and meropenem significantly reduces viable cell counts of both species, underscoring the clinical importance of anti-biofilm combination strategies.

## 4 Pathogenesis and risk factors of HD-CRBSI

The pathogenesis of CRBSI in HD patients involves intricate interactions among microbial factors, catheter properties, and host defenses. Clinically, two primary routes lead to CRBSI: the extraluminal (or percutaneous) route, where skin flora migrate along the external catheter surface from the insertion site or tunnel (in the case of tunneled catheters); this underscores the critical importance of optimal skin antisepsis during insertion, meticulous exit-site care, and the use of secure, antimicrobial dressings. The intraluminal (or hub) route, where microorganisms contaminate the catheter hub or lumen during connection or manipulation, highlighting the necessity of strict aseptic technique for all catheter accesses and the potential value of antimicrobial catheter lock solutions to prevent intraluminal biofilm formation. Both routes can culminate in microbial colonization of the catheter surface, where the formation of a structured biofilm becomes the central mechanism facilitating persistent infection (Colombari et al., 2021). Biofilm formation involves the adhesion of microorganisms to the catheter surface and secretion of extracellular polymeric substances (EPS), progressively establishing complex architectures. In the context of HD, biofilm development is closely linked to repeated catheter accesses for dialysis sessions, which can introduce pathogens via the intraluminal route, and to the chronic presence of a foreign body, which provides a substrate for colonization originating from either route.​ Understanding these pathways directly informs the multi-modal prevention strategies, such as care bundles, that target both the extraluminal and intraluminal sources of infection. These biofilm structures not only provide physical protection for microbes but also create specialized microenvironments that enhance the survival and persistence of antibiotic-resistant strains (Almeida et al., 2022; Romero et al., 2025). The structural composition of HD catheters offers a substrate for bacterial adhesion, with coagulase-negative staphylococci being particularly associated with catheter colonization (Blanco-Di Matteo et al., 2022).

Within biofilms, bacteria utilize quorum sensing systems for intercellular communication and coordinated gene expression. Biofilm maturation creates a physical barrier of extracellular polymeric substances that shields bacteria from therapeutic antibiotic concentrations, directly explaining why *in vitro* susceptibility often fails to predict *in vivo* response (Li et al., 2023). The subsequent dispersion phase releases planktonic cells into the bloodstream, corresponding to the recurrent fevers, chills, and positive blood cultures observed clinically (Cuevas et al., 2020).

**Figure 1:**
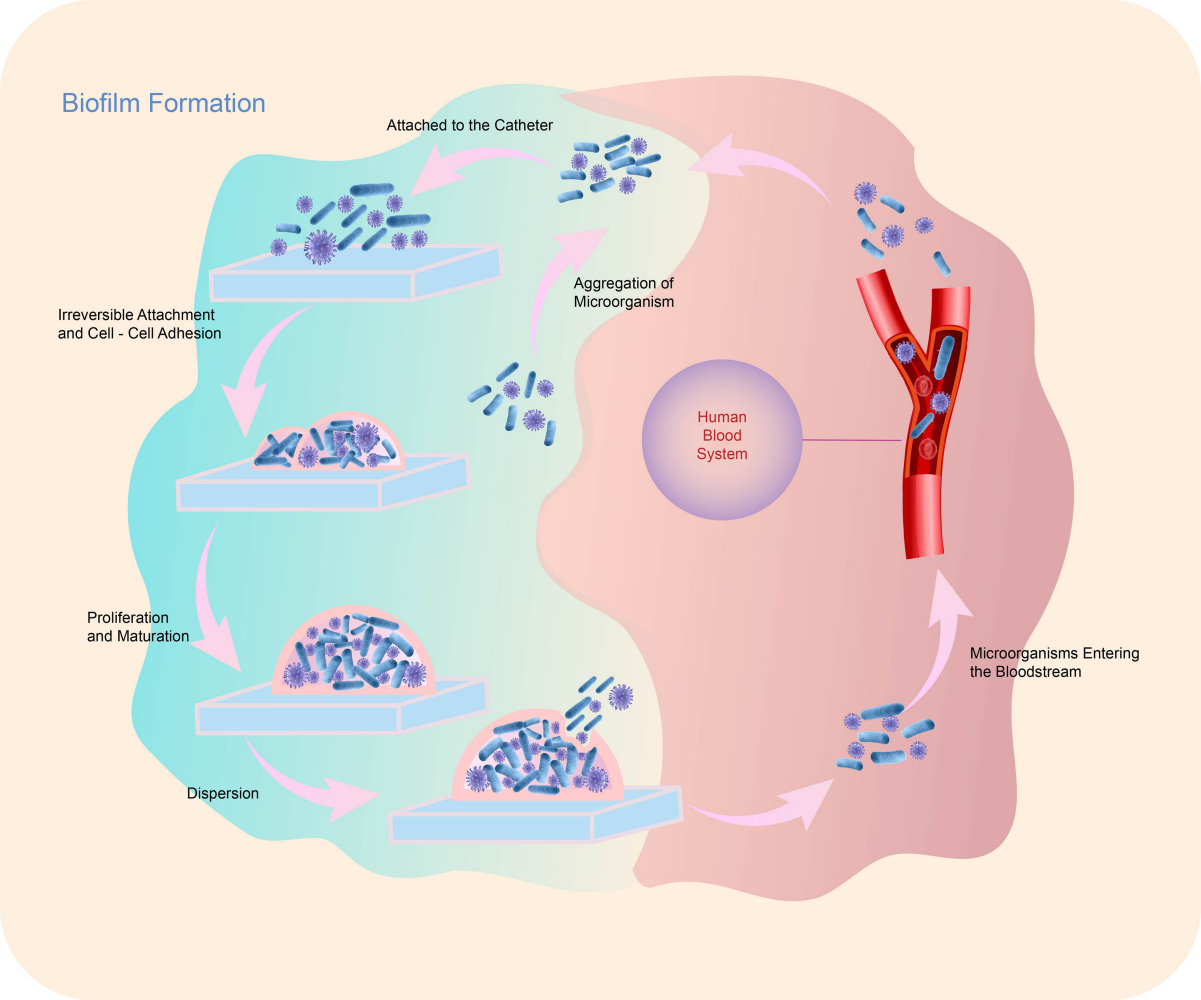
Mechanism of biofilm formation in central venous catheter-related bloodstream infections among hemodialysis patients. This schematic illustrates the pathogenesis of persistent bloodstream infections due to catheter biofilm formation in hemodialysis patients. The process begins with microbial adhesion (Attached to the Catheter), followed by irreversible attachment and intercellular adhesion via matrix secretion (Irreversible Attachment and Cell-Cell Adhesion). The biofilm then proliferates and matures (Proliferation and Maturation), evolving into a drug-resistant, immune-evasive complex. Subsequent dispersion (Dispersion) releases planktonic cells or fragments into the bloodstream (Microorganisms Entering the Bloodstream), causing bacteremia. The dispersed units in the bloodstream can re-disseminate to the catheter, aggregate with catheter-colonizing microbes (Aggregation of Microorganisms) and recolonize, restarting the “adhesion–attachment–proliferation/maturation–dispersion” cycle, which underlies chronic, recurrent, and refractory infections.

In summary, the pathogenesis of HD-CRBSI is a cyclical process driven by biofilm formation, where microbial adhesion, attachment, proliferation/maturation and dispersion create a self-perpetuating loop of colonization and infection. This central mechanism is illustrated in **Figure 1**. This biofilm-driven cycle explains why catheter retention in the setting of established biofilm infection frequently results in persistent or recurrent CRBSI, and why catheter removal remains the most reliable intervention for source control.

Multiple risk factors contribute to the pathogenesis and progression of CRBSI. HD patients exhibit specific vulnerability factors that elevate CRBSI susceptibility. A cross-sectional study conducted at a tertiary hospital in Ghana revealed a remarkably high CRBSI prevalence rate of 34.2% among HD patients, with coagulase-negative staphylococci being the predominant pathogens (Opoku-Asare et al., 2023). Significant predictors included male gender, shorter dialysis vintage, and diabetes-mediated end-stage renal disease. Patient-related determinants encompass advanced age, comorbidities, immunocompromised status, and metabolic-nutritional impairments. Treatment-associated variables involve catheter dwell time, insertion site characteristics, catheter type selection, and procedural frequency.

Patient-related risk factors constitute the intrinsic foundation for infections, demonstrating multidimensional and cumulative mechanisms of action. HD patients frequently present with comorbidities such as cardiovascular diseases and anemia, which may compromise immune function and heighten infection susceptibility. Advanced age represents a prominent risk factor, where natural immune senescence, escalating comorbidity burden, and progressive vascular deterioration collectively impair host defense mechanisms. Underlying conditions including diabetes mellitus and chronic kidney disease not only cause primary damage to microcirculatory structure and function, but also amplify infection susceptibility through chronic inflammatory states and immune dysregulation (Zhou et al., 2023). Nutritional-metabolic disorders, particularly hypoalbuminemia serving as a marker of systemic inflammation and protein-energy wasting, directly impair immunocyte functionality and tissue repair capacity (Bnaya et al., 2023). Patients with obesity, especially those exhibiting BMI≥40, demonstrate significantly elevated risks of intravascular catheter-related infections. These interrelated factors collectively establish the intrinsic predisposing background for CRBSI development and progression.

Catheter-related factors play a pivotal role in the pathogenesis of CRBSI. Prolonged catheter indwelling duration significantly elevates infection risk (Buetti et al., 2021). Studies demonstrate that catheters remaining *in situ* beyond 21 days exhibit substantially higher CRBSI incidence compared to short-term indwelling catheters (Pitiriga et al., 2025). The anatomical insertion site selection directly correlates with infection risk, with femoral vein catheterization (FVC) constituting an independent risk factor for CRBSI recurrence. Differential infection risks exist among catheter types; comparative studies reveal that peripherally inserted central catheters (PICC) and CVC may manifest distinct infection risk profiles contingent upon specific clinical scenarios (Lang et al., 2025). Renal replacement therapy modalities similarly influence infection potential, wherein continuous renal replacement therapy (CRRT) potentially augments catheter-related infection risks relative to intermittent HD (Parienti et al., 2010).

The surface characteristics of catheter materials directly govern microbial adhesion and biofilm formation capacity. Conventional materials facilitate bacterial colonization, whereas novel antimicrobial-coating materials effectively mitigate this process. Phytosynthesized zinc oxide nanoparticle coatings (ZnO NPs) create hydrophilic surfaces that effectively suppress biofilm formation by major pathogens including Staphylococcus aureus, Escherichia coli, and Pseudomonas aeruginosa, demonstrating inhibition rates of 90-97% (Malhotra et al., 2023). Emerging evidence indicates that microbiome alterations may modulate CRBSI susceptibility. Gut microbiota dysbiosis potentially promotes pathogen translocation, thereby increasing bloodstream infection risks (Liu et al., 2025). This represents an emerging research direction with clinical interventions that remain underdeveloped, yet its association with systemic inflammatory conditions offers novel insights for future preventive strategies. Additionally, interspecies pathogen interactions may influence infection progression, as evidenced by bacteria-fungal synergistic interactions within dual-species biofilms potentially enhancing antimicrobial resistance profiles (Kurakado et al., 2025). These findings provide novel conceptual frameworks for CRBSI prevention through microbiome modulation strategies. The interplay of patient-specific vulnerabilities, catheter-related characteristics, and pathogen-specific factors constitutes the multifactorial risk landscape for HD-CRBSI. A consolidated summary of these key risk elements is provided in **Table 1**.

**Table 1:** Comprehensive risk factors assessment for hemodialysis catheter-related bloodstream infections.

Key evidence from literature	Mechanism	Reference	Evidence strength
Catheter Hub Handling	Failure of hub disinfection before dialysis allows intraluminal bacterial entry.	Kotwal et al., 2022	Strong
Repeated Access During Dialysis Sessions	Repeated opening of the closed system for dialysis access increases contamination risk.	Almeida et al., 2022	Strong
BMI ≥ 40 (Obesity)	Independent risk factor causing direct immunological injury.	Buetti et al., 2021	Strong
Indwelling time >21 days	Prolonged catheterization significantly increases infection risk (approximately 3.5-fold).	Pitiriga et al., 2025	Moderate
Femoral vein catheterization	Independent risk factor; higher infection risk vs IHD.	Pitiriga et al., 2025	Moderate
Diabetes→ESRD	CKD and diabetes cause microcirculatory impairment, chronic inflammation, and immune dysregulation.	Buetti et al., 2021	Moderate
Hypoalbuminemia (<3.5g/dL)	Marker of systemic inflammation and protein-energy wasting; impairs immune function and tissue repair.	Bnaya et al., 2023	Moderate
Advanced age (70 y)	Immune senescence, comorbidity accumulation, and vascular deterioration.	Bnaya et al., 2023	Moderate
Male gender	Reported as an independent predictor in selected studies.	Bnaya et al., 2023	Moderate
Biofilm Formation	EPS secretion creates a protective microenvironment, shielding bacteria from antibiotics and host immunity.	Colombari et al., 2021	Weak
Quorum Sensing	Bacterial intercellular communication enhances coordinated antimicrobial resistance.	Li et al., 2023	Weak
Gut Microbiota Dysbiosis	Promotes pathogen translocation and elevates CRBSI risks.	Liu et al., 2025	Weak

BMI, Body Mass Index; ESRD, End-Stage Renal Disease; CKD, Chronic Kidney Disease; EPS, Extracellular Polymeric Substances; IHD, Intermittent Hemodialysis; CRBSI, Catheter-Related Bloodstream Infections.

The pathogenesis of HD-CRBSI represents a complex process primarily governed by biofilm formation with multifactorial contributions. Pathogens leverage biofilms to acquire robust drug resistance and persistence, while host factors, catheter characteristics, and therapeutic interventions collectively establish the intrinsic foundation and extrinsic conditions conducive to infection. A profound comprehension of this multidimensional interaction network is essential for developing precise, comprehensive prevention and control strategies.

## 5 Strategies and interventions for preventing HD-CRBSI

### 5.1 Optimization of catheter insertion and maintenance techniques

Optimization of catheter insertion and maintenance techniques constitutes the fundamental component in preventing CRBSI. Strict aseptic technique serves as the cornerstone for preventing pathogen invasion, with all venous catheter manipulations requiring adherence to the no-touch sterile technique (Mistry et al., 2025). During catheter placement, rigorous compliance with sterile operating protocols represents the core prerequisite, with preferential selection of the right internal jugular vein recommended to avoid the elevated risks associated with femoral vein catheterization. The application of ultrasound-guided technology has significantly enhanced insertion precision and safety while effectively reducing puncture-related tissue damage. A study focusing on severely traumatized patients demonstrated that ultrasound-guided axillary venous central catheterization (UAVC) achieved relatively high success rates without immediate complications, though the optimal catheter tip positioning rate reached only 29.8% (Gu et al., 2025). These findings indicate that while the technique itself proves safe and effective, further refinement remains necessary regarding operational standardization and implementation.

Post-cannulation standardized maintenance is equally critical, necessitating establishment of a tripartite healthcare provider-patient collaborative management system. Healthcare personnel must rigorously implement hand hygiene and aseptic techniques while standardizing dressing change protocols, whereas patients require enhanced self-care education and timely reporting of abnormalities. A user-centered design study demonstrated that novel patient engagement models play pivotal roles in improving therapeutic participation and complication prevention. This framework achieves synergistic benefits through personalized care, strengthened interpersonal connectivity, competency-building education, and preservation of patient autonomy—thereby establishing positive behavioral reinforcement between “post-dialysis clinical improvement” and self-management practices for complication mitigation (Willis et al., 2021). Furthermore, strict adherence to catheter dwell-time minimization principles remains essential, with prompt removal upon clinical obsolescence (Buetti et al., 2023).

The method of catheter securement significantly impacts infection risk. Sutureless fixation devices Geometric Retention Interface Platform - Locking Organ fixation Kit (GRIP-LOK) demonstrate superior performance compared to traditional skin sutures (Fujimoto et al., 2021). A single-center retrospective cohort study conducted at Kanazawa Medical University Hospital involving 211 non-tunneled HD catheters (NTHC) revealed that the GRIP-LOK group exhibited a CRBSI incidence rate of 1.44 per 1000 catheter-days, significantly lower than the suture group’s rate of 5.25 per 1000 catheter-days. Additionally, the exit-site infection incidence decreased to zero (compared to 5.43 per 1000 catheter-days in the suture group), establishing GRIP-LOK as a safer practice option for HD catheter securement (Fujimoto et al., 2021). Another crucial consideration involves dressing selection—chlorhexidine gluconate-impregnated dressings demonstrate reduced catheter-associated bloodstream infection frequency per 1000 patient-days when compared to standard polyurethane dressings (Ullman et al., 2015).

Recent years have witnessed the application of innovative technologies in catheter insertion and maintenance procedures. Near-infrared vascular imaging devices have demonstrated higher first-attempt success rates for peripheral intravenous catheter placement in ICU patients (Ferrier et al., 2025). Wireless intracardiac electrocardiogram (IC-ECG) technology provides real-time intraoperative feedback with high sensitivity and specificity, reducing reliance on radiographic confirmation while significantly lowering complication risks and improving procedural efficiency (Gianazza et al., 2025). These auxiliary tools not only enhance operational effectiveness but also minimize tissue trauma and infection risks associated with multiple insertion attempts.

Although these interventions demonstrate promising potential, some large-scale studies have yielded unexpected findings. The REDUCCION trial—a multifaceted intervention study employing stepped-wedge cluster randomization across 37 Australian renal service centers—revealed no significant reduction in CRBSI incidence (Kotwal et al., 2022). This negative outcome warrants critical analysis. It may not inherently indicate the ineffectiveness of the bundled interventions themselves, but could reflect challenges in implementation fidelity across diverse clinical settings. Potential factors include variability in staff compliance, insufficient training or resources to sustain all intervention components uniformly, or the presence of unmeasured confounding variables specific to each center. The result underscores a crucial lesson for implementing complex care bundles: their success is contingent not only on the evidence-based components but equally on robust strategies for ensuring consistent adoption, monitoring adherence, and adapting to local contextual barriers. Therefore, future initiatives should integrate rigorous process evaluation alongside outcome assessment to distinguish between intervention failure and implementation failure. This surprising outcome suggests that multifaceted CRBSI prevention strategies may not achieve anticipated effectiveness across all clinical settings, highlighting the challenges and complexities inherent in translating evidence-based practices to diverse healthcare environments.

### 5.2 Antibacterial-coated and material-innovated catheters

Antimicrobial-coated catheters represent a significant technological innovation for preventing CRBSI. By incorporating antimicrobial agents onto catheter surfaces, these devices effectively inhibit pathogen colonization and biofilm formation. Research demonstrates that combining nitric oxide (NO) with 70% isopropyl alcohol through the integration of S-Nitroso-N-Acetylpenicillamine (SNAP) into hydrophilic-modified polydimethylsiloxane (PDMS-PEO) sponges can substantially reduce bacterial viability on needleless connectors (Goodman et al., 2025).

Emerging permanent coating technologies demonstrate promising clinical potential. LubriShield, a novel permanent coating applied uniformly to both luminal and exterior surfaces of indwelling urinary catheters, effectively prevents biofilm formation by pathogens without releasing active agents. This superhydrophilic coating incorporates proprietary antifouling ligands that significantly inhibit biofilm formation by uropathogens in artificial urine medium for up to 14 days.

Pathogen-specific antimicrobial strategies continue to evolve. In murine catheter-associated urinary tract infection models, protein subunit vaccines targeting Acinetobacter baumannii adhesin Abp2D demonstrated significant reduction in bacterial titers (Timm et al., 2023). Passive immunization experiments revealed that Abp2D immune serum could transfer immunity to naive mice and inhibit bacterial binding to fibrin-coated catheters.

### 5.3 Catheter lock solution strategies and antimicrobial lock therapy

Antibiotic Lock Therapy (ALT) serves as an effective approach for preventing and treating CRBSI, employing high-concentration antibiotic solutions within the catheter lumen maintained for sufficient dwell time to eradicate or suppress biofilm-embedded pathogens (Blanco-Di Matteo et al., 2022). Studies confirm ALT’s successful application in CRBSI cases where central venous catheter removal is clinically contraindicated (Güneş et al., 2025). A meta-analysis of RCTs demonstrated that antimicrobial lock solutions reduced CRBSI risk by 69%, exit-site infections by 32%, without significantly impacting catheter failure from non-infectious complications (Zacharioudakis et al., 2014). The LOCK IT-100 trial further validated the efficacy of taurolidine/heparin lock solutions in preventing CRBSI among HD patients. Novel enhanced lock solutions continue to emerge. A bifunctional low-molecular-weight heparin-coated silver nanoparticle formulation exhibits dual anticoagulant and antimicrobial/anti-biofilm properties (Meher and Poluri, 2022). The LOCK-IT-100 trial, a multicenter phase 3 randomized controlled trial in 795 adults receiving maintenance hemodialysis, further demonstrated that a taurolidine/heparin lock solution (taurolidine 13.5 mg/mL + heparin 1000 U/mL) significantly reduced the risk of CRBSI compared with heparin alone (HR 0.28; 95% CI 0.13–0.59), with the trial terminated early for high statistical significance (Agarwal et al., 2023). These formulations demonstrate twofold enhanced anticoagulant activity, coupled with high efficacy against mono- and polymicrobial biofilm inhibition/eradication while maintaining hemocompatibility.

Beyond lock solutions, innovative catheter materials themselves are a key preventive frontier. Recent research focuses on coatings specifically for hemodialysis catheters. Heparin-coated catheters aim to reduce thrombus formation, a known nidus for infection. More directly, antimicrobial coatings impregnated with agents like silver ions or chlorhexidine have been developed. A Cochrane systematic review reported that antimicrobial-impregnated CVC significantly reduced CRBSI risk compared to standard catheters; however, the included studies were heterogeneous, most were not conducted specifically in HD populations, and the cost-effectiveness of routine use in maintenance hemodialysis remains to be established (Wang et al., 2018).

The selection of catheter lock solutions requires comprehensive consideration of antimicrobial efficacy, safety, and drug resistance risks. Although antibiotic lock solutions demonstrate effectiveness, their potential contribution to the selection of resistant strains necessitates cautious application. Daptomycin and teicoplanin lock solutions exhibit significantly superior performance over standard therapy in eliminating Coagulase-negative staphylococci (CoNS) biofilms within Tunneled Central Venous Catheter (TCVC). However, repeated teicoplanin use may accelerate resistance development, while prolonged vancomycin administration could potentially reduce resistance to other antibiotics through the “seesaw effect.” (Blanco-Di Matteo et al., 2023) biotic alternatives such as citrate and ethanol are gaining increasing attention due to their antimicrobial efficacy coupled with reduced antibiotic selection pressure (Wang and Sun, 2022). An innovative formulation combining trimethoprim, ethanol, and calcium disodium EDTA demonstrated a 4.56-fold reduction in CRBSI incidence compared to heparin locks (Rijnders et al., 2019). A descriptive cohort study reported a CRBSI rate of 2.3 per 1000 catheter-days among adult home parenteral nutrition (HPN) patients using citrate locks (Leong et al., 2025). Notably, PICC showed higher infection rates than TCVC, suggesting catheter type as a potential determinant of lock efficacy. Retrospective analysis revealed ELT achieved a remarkable 19-fold CRBSI reduction in adult HPN patients, establishing its clinical utility for TCVC while highlighting the need for prospective RCTs to optimize ethanol concentration, dosing, and exposure frequency (Gundogan et al., 2020).

Researchers developed a novel non-antibiotic antimicrobial lock solution (SNACET) targeting CRBSI prevention, which exerts bactericidal effects via NO release capable of biofilm penetration while circumventing resistance concerns (Kumar et al., 2021). This innovation provides an alternative approach to overcome the resistance development and permeability limitations inherent to antibiotic locks. Multicenter clinical studies remain imperative to establish optimal concentrations, compatibility profiles, and long-term safety parameters across lock solution categories, thereby generating robust evidence for standardized catheter management protocols.

From an economic perspective, antimicrobial catheter lock solutions demonstrate favorable cost-effectiveness characteristics. Probabilistic analysis indicates that in HD settings with a willingness-to-pay threshold of $50,000, antimicrobial locks exhibit a 96.24% probability of being more cost-effective than heparin locks (Pliakos et al., 2019). A base-case analysis revealed that preventing each CRBSI episode in HD environments yields cost savings of $68,721.03.

### 5.4 Bundled interventions and clinical practice

Bundled interventions comprehensively reduce CRBSI risk by combining multiple evidence-supported practices into standardized protocols. Integrated care bundles addressing multiple aspects of catheter management have demonstrated significant effectiveness in decreasing infection rates. Implementation of a six-component dialysis event prevention bundle showed substantial reductions in intravenous antimicrobial use, positive blood cultures, and vascular access site inflammation (Alhulays et al., 2024) Neonatal ICU implementing updated international guideline-based bundles (encompassing aseptic techniques, material replacement, and catheter maintenance) achieved a CRBSI reduction from 8.4 to 1.8 cases per 1000 catheter-days, with catheter-related complications decreasing from 47 to 10 cases. Nurse-led implementation of evidence-based bundles demonstrated notable efficacy in CAUTI prevention, yielding a 38% reduction in infection risk and 11% decrease in inappropriate catheter utilization rates (Riley et al., 2025).

Quality improvement methodologies have been successfully applied to CRBSI prevention. The Lean Six Sigma approach utilizing the Define-Measure-Analyze-Improve-Control framework significantly reduced CRBSI rates from 12.79 to 2.32 cases per 1000 catheter-days (Feng et al., 2024). This methodology employed value stream mapping, fishbone diagrams, and root cause analysis to identify underlying CRBSI contributors, with its demonstrated effectiveness in non-ICU settings suggesting broader applicability for HD patient care.

Successful implementation of bundled interventions requires multidisciplinary team collaboration and comprehensive staff training. Educational programs, clinical decision-support tools, and multidisciplinary approaches have proven effective in reducing unnecessary blood and urine culture testing. Furthermore, structured training, infection control measures, and policy-level support are essential for widespread adoption and outcome improvement in resource-limited settings (Saha et al., 2025).

The implementation science framework plays a pivotal role in disseminating bundled interventions. Current research demonstrates that such frameworks bridge the “evidence-practice gap” through structured pathways, proving particularly critical for deploying complex interventions (Huybrechts et al., 2021). A scoping review of implementation frameworks, strategies, and outcomes for central venous access device (CVAD) practices revealed “quality improvement” as the most prevalent framework, with clinician education, audit-feedback systems, and care bundles being the most frequently cited implementation strategies (Comber et al., 2025).

### 5.5 Optimization of diagnostic and management pathways

Optimization of diagnostic and management pathways is critical for both prevention and early intervention of CRBSI. Diagnostic stewardship serves to improve laboratory testing utilization patterns by enhancing patient care while reducing unnecessary tests. This strategy encompasses three key aspects: minimizing redundant testing, maximizing the clinical utility of available diagnostic methods, and preventing overdiagnosis of healthcare-associated infections.

CRBSI in HD patients is a leading cause of high mortality. However, due to nonspecific symptoms and the time-consuming nature of conventional blood cultures (6–72 hours), clinicians often rely on empirical broad-spectrum antibiotics, increasing the risk of antimicrobial resistance (Nguyen et al., 2017). Novel diagnostic methods show promise in improving CRBSI detection. Real-time polymerase chain reaction (rt-PCR) demonstrates superior diagnostic performance, with universal bacterial 16S primers achieving 100% sensitivity and 78% specificity within 3.5 hours. Studies indicate that rt-PCR-guided diagnostics can reduce empirical treatment for Gram-positive cocci, significantly enhancing antibiotic stewardship (Acquier et al., 2023). RT-PCR exhibits rapid, highly sensitive diagnostic potential for suspected HD-CRBSI, improving antimicrobial management and lowering resistance risks, though further multicenter validation is needed to standardize the technique.

Inflammatory biomarkers provide additional diagnostic utility for early CRBSI detection. The systemic inflammatory response index (SIRI) demonstrates particularly strong diagnostic performance. Other biomarkers, including neutrophil-to-lymphocyte ratio, platelet-to-lymphocyte ratio, and C-reactive protein-to-albumin ratio, also exhibit significant diagnostic value (Yang et al., 2022). Notably, Gram-negative infections show markedly higher SIRI values compared to Gram-positive infections, potentially guiding empirical antibiotic selection.

Artificial intelligence technologies, particularly machine learning, demonstrate promising potential for early detection of vascular catheter-associated infections (VCAI). A study employing gradient boosting models achieved 82.5% balanced accuracy with 67% sensitivity, indicating their clinical utility for VCAI prewarning (Spaimoc et al., 2025). Furthermore, generative AI showed 95.2% sensitivity and 76.2% specificity in CAUTI detection, with specificity improving to 90% after expert validation (Alshanqeeti et al., [[NoYear]]).

Standardized diagnostic criteria are critical for enabling accurate monitoring of vascular catheter-associated infections and facilitating valid inter-institutional comparison of infection rates. However, significant standardization gaps persist in current practice. A multicenter evaluation revealed that most laboratories fail to systematically track key quality metrics like single-blood-culture rates or blood-culture positivity rates, while notable inconsistencies exist in defining bloodstream infection cases (Fabre et al., 2025). These variations in definitions and reporting frameworks substantially compromise data comparability and accurate assessment of intervention effectiveness. Diagnostic standardization is further complicated by difficulties in establishing uniform CRBSI definitions across healthcare settings. Research demonstrates that CRBSI reporting achieves high concordance when standardized endpoint definitions are employed. Exit-site infections show the greatest consistency, while other infection categories exhibit more classification variability. These findings suggest that consistent endpoint definitions enable reliable benchmarking without requiring independent clinical adjudication (Catiwa et al., 2024). Therefore, establishing and widely adopting unified bloodstream infection definitions with standardized reporting protocols would markedly improve the accuracy of infection rate statistics, enhance reliability of clinical outcome analyses, and provide robust evidence for evaluating prevention and treatment strategies (Averbuch et al., 2025).

An effective defense against HD-CRBSI requires a multi-layered strategy encompassing technical, technological, and systematic interventions. An overview of the core prevention strategies, supported by key evidence, is consolidated in **Table 2**.

**Table 2:** Comprehensive prevention strategies for Hemodialysis Catheter-Related Bloodstream Infection (HD-CRBSI).

Strategy	Specific measures	Key points	Reference
Standard clinical practices			
Catheter Hub Care	Scrub-the-hub with antiseptic before each access; antiseptic-impregnated port protectors; closed-system connectors	Mandatory disinfection before every connection prevents intraluminal contamination	Agarwal et al., 2023
Aseptic Connection/Disconnection	Standardized ANTT for HD line connection and disconnection; sterile gloves and masks during catheter manipulation	Minimizes contamination during the most frequent breach of the closed system	Agarwal et al., 2023
Catheter Insertion and Maintenance	Ultrasound guidance; maximal sterile barrier precautions; chlorhexidine skin antisepsis; sutureless securement devices; chlorhexidine-impregnated dressings	Core bundle components with strong evidence base	Lazarus et al., 2023
Staff Training and Care Bundles	Standardized insertion and maintenance checklists; regular competency assessment; audit and feedback; multidisciplinary collaboration	Ensures consistent implementation; addresses compliance variability and educational gaps	Alhulays et al., 2024
Catheter Lock Solutions	Citrate (>0.2%) or taurolidine-based locks; avoidance of heparin-only locks where possible	Non-antibiotic alternatives reduce resistance risk; supported by meta-analyses and RCT	Zacharioudakis et al., 2014
Minimizing Catheter Dwell Time	Daily assessment of catheter necessity; prompt removal when no longer indicated; early planning for permanent vascular access	Single most effective strategy for long-term CRBSI reduction	Buetti et al., 2023
Emerging and Experimental Strategies			
Antimicrobial-Coated Catheters	Chitosan/copper composite coatings; heparin-coated catheters	Preclinical and early clinical data promising; HD-specific efficacy data still limited	Gu et al., 2022
Novel Biomaterials	Nitric oxide-releasing coatings; superhydrophilic permanent coatings; zinc oxide nanoparticles	Preclinical or early experimental stage; further validation required	Malhotra et al., 2023
Pathogen-Specific Strategies	Anti-adhesin vaccines (e.g., Abp2D); bacteriophage-enzyme composite coatings	Experimental; no human data available	Timm et al., 2023
AI-Assisted Monitoring	Machine learning-based risk prediction; automated surveillance algorithms	Retrospective data promising; requires prospective multicenter validation	Wójcicki et al., 2025

HD-CRBSI, Hemodialysis Catheter-Related Bloodstream Infection; ANTT, Aseptic Non-Touch Technique; CRBSI, Catheter-Related Bloodstream Infection; HD, Hemodialysis; RCT, Randomized Controlled Trial.

## 6 Treatment and management for HD-CRBSI

### 6.1 Clinical assessment and classification

A structured clinical approach is essential for managing HD-CRBSI. Initial management should be guided by promptly categorizing the infection, which dictates the urgency and setting of treatment.

#### 6.1.1 Uncomplicated vs. complicated CRBSI​

CRBSI should be stratified into uncomplicated and complicated infections. Uncomplicated CRBSI defined by infection limited to the bloodstream, with prompt defervescence and clearance of bacteremia (e.g., within 48-72 hours) after initiating appropriate antibiotics, in a patient who is hemodynamically stable and has no evidence of metastatic infection (e.g., endocarditis, septic thrombosis) or deep-seated catheter involvement (tunnel infection). Complicated CRBSIis characterized by persistent fever or bacteremia (>72 hours) despite appropriate therapy, HD instability (sepsis or septic shock), or the presence of metastatic infectious complications.

#### 6.1.2 Outpatient vs. inpatient management

The treatment setting depends on infection severity and patient stability. Patients with uncomplicated CRBSI who are clinically stable may be managed in an outpatient setting with close monitoring, provided they can receive appropriate parenteral antibiotics (often administered at the dialysis center) and adhere to follow-up. Hospitalization is mandatory for those with complicated CRBSI, signs of severe sepsis, or for patients who cannot be managed safely as outpatients.

#### 6.1.3 Anatomical site of infection​

It is critical to differentiate the primary focus. Exit-site infection involves inflammation confined to the catheter skin exit point. Tunnel infection refers to inflammation extending >2 cm along the subcutaneous catheter track, often with tenderness. Systemic CRBSI(the focus of this section) is confirmed by positive blood cultures with the catheter as the source. Exit-site or tunnel infections may precede or coexist with CRBSI and often necessitate catheter removal, especially if there is purulence or the infection progresses despite therapy.

**Figure 2:**
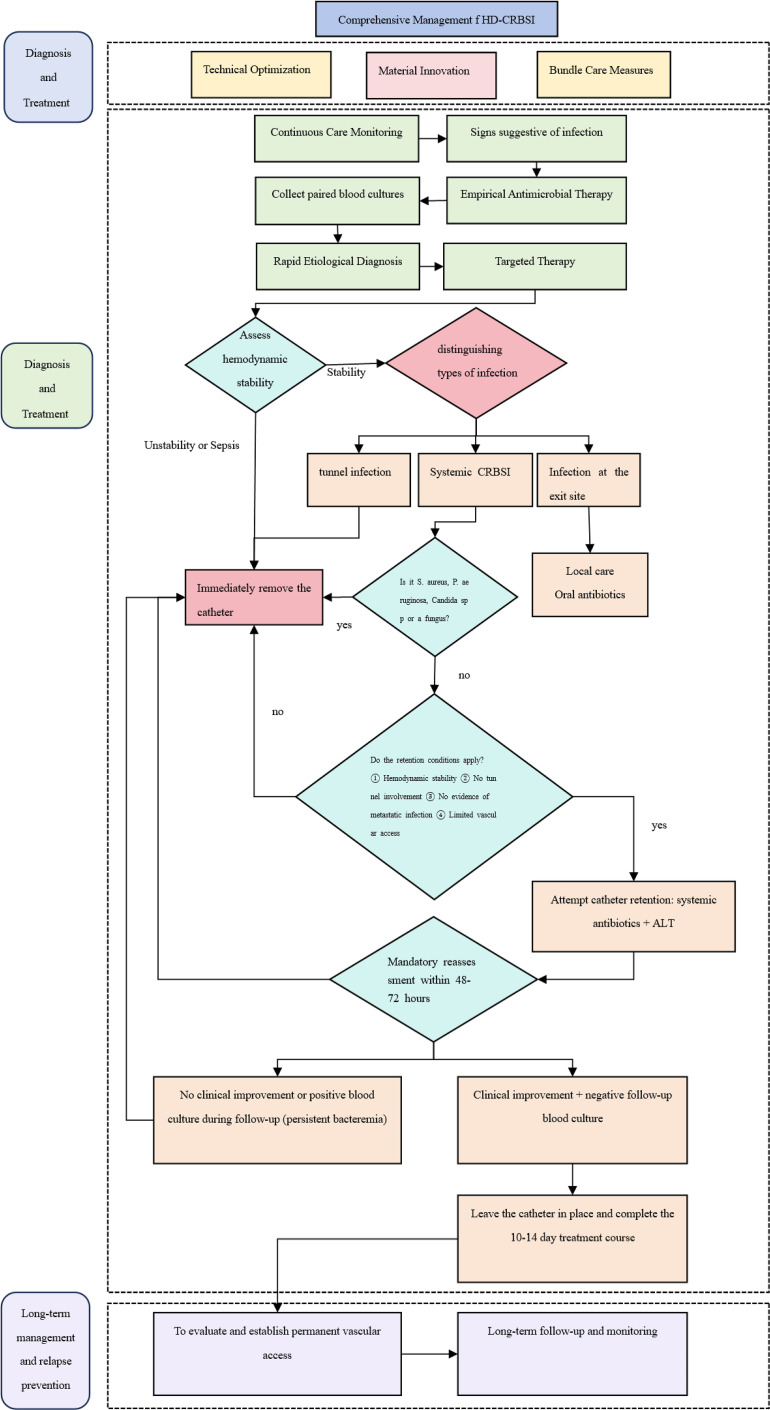
Comprehensive management protocol for catheter-related bloodstream infections in hemodialysis patients. ALT, Antimicrobial Lock Therapy; CRBSI, Catheter-Related Bloodstream Infection; HD, Hemodialysis; S. aureus, Staphylococcus aureus; P. aeruginosa, Pseudomonas aeruginosa; Candida sp., Candida species.

This initial assessment framework, summarized in **Figure 2**, directly informs subsequent decisions regarding empiric therapy, catheter management, and treatment duration.

### 6.2 Empirical antimicrobial therapy

Empiric therapy should be initiated immediately upon clinical suspicion of CRBSI following paired blood culture collection, without awaiting microbiologic results. Selection of the initial regimen requires comprehensive consideration of the patient’s clinical severity, history of colonization or infection with resistant pathogens, and local epidemiological data.

For HD patients, empirical regimens must provide coverage against both Gram-positive bacteria (particularly methicillin-resistant Staphylococcus aureus, MRSA) and Gram-negative organisms (including Pseudomonas aeruginosa, P. aeruginosa). This is necessitated by the significant prevalence of MRSA and P. aeruginosa in the causative pathogens of CRBSI among this patient population. A standard protocol involves vancomycin combined with an anti-pseudomonal β-lactam antibiotic (such as ceftazidime or cefepime) (Surapat et al., 2020).

This combined regimen is particularly appropriate in settings with a high prevalence of MRSA. However, therapeutic choices must be tailored to local epidemiology. In regions with low MRSA prevalence where methicillin-susceptible *S. aureus (MSSA*) predominates, a β-lactam such as cefazolin may be a superior choice for Gram-positive coverage due to its faster bactericidal activity and potentially better efficacy against MSSA compared to vancomycin. This underscores the critical importance of basing empirical therapy decisions on current, facility-specific antibiogram data to optimize outcomes and avoid unnecessary use of broad-spectrum agents.

Vancomycin serves as the cornerstone therapeutic agent for MRSA and most Gram-positive bacterial coverage. However, its pharmacokinetic profile is notably altered in HD patients, as the drug undergoes significant clearance during dialysis sessions. Consequently, the standard protocol involves administering the dose post-dialysis, accompanied by routine trough concentration monitoring to ensure therapeutic efficacy while mitigating potential toxicity (Creţu et al., 2025). For patients with Gram-negative bacterial infections (including Pseudomonas aeruginosa), common options include ceftazidime or cefepime. In specific scenarios (such as septic shock or high-risk patients for antibiotic resistance), combination therapy with an aminoglycoside (e.g., gentamicin) may be employed (Abutaha et al., 2022).

For critically ill patients or those with documented history of multidrug-resistant organism colonization/infection, an intensified regimen combining vancomycin with an anti-pseudomonal cephalosporin (ceftazidime/cefepime) should be implemented. In select extremely high-risk scenarios, carbapenem-class agents (e.g., meropenem) may be warranted (Sivori et al., 2025). For patients with risk factors for Candida infection (such as prolonged broad-spectrum antibiotic use or femoral vein catheterization), empirical addition of an echinocandin antifungal agent should be considered.

### 6.3 Targeted antimicrobial therapy

Based on confirmed pathogen identification and susceptibility results, targeted therapy should follow precision medicine principles, employing narrowly-spectrum antimicrobials with specific activity. For coagulase-negative staphylococci, methicillin-susceptible strains may be de-escalated to cefazolin, with treatment duration shortened to 5-7 days after catheter removal for confirmed, uncomplicated bacteremia where the catheter has been removed, as supported by clinical guidelines and evidence or extended to 10-14 days if the catheter is retained. Upon diagnosis of Staphylococcus aureus bacteremia, catheter removal is strongly recommended, along with selection of the most effective antibiotics: cefazolin (superior to vancomycin) for MSSA, and vancomycin or daptomycin for MRSA (Farrington and Allon, 2019; Apata et al., 2021). Daptomycin holds particular importance as an alternative for MRSA when vancomycin is unsuitable due to resistance, allergy, or concerns for nephrotoxicity. The pharmacokinetics of many antibiotics, including vancomycin and aminoglycosides, are significantly altered in patients on hemodialysis due to substantial clearance of the drug during the dialysis session. Therefore, to ensure adequate therapeutic exposure, these agents should be administered after the completion of a dialysis session. For HD patients with uncomplicated MRSA bacteremia, the recommended vancomycin dosing regimen typically includes a loading dose of 15-25 mg/kg (based on actual body weight)​ administered after a dialysis session to rapidly achieve therapeutic levels. This is followed by maintenance dosing, which is also administered post-dialysis. The maintenance dose is individualized based on therapeutic drug monitoring (TDM).​ For vancomycin, TDM of trough concentrations is mandatory to ensure efficacy and minimize toxicity; the target trough level for serious MRSA infections is generally 15-20 mg/L. Trough levels should be checked just prior to a subsequent dose (pre-dialysis) to guide dose adjustments (Ramirez-Osorio et al., 2024).

For uncomplicated bacteremia, antimicrobial therapy should be administered for at least 14 days following catheter removal; if deep-seated infection is present, treatment duration should be extended to 4-6 weeks.

For Gram-negative bacilli without drug resistance, third-generation cephalosporins, fluoroquinolones, or β-lactam/β-lactamase inhibitor combinations should be selected based on antimicrobial susceptibility testing. In cases of Pseudomonas aeruginosa infection, catheter removal is strongly recommended, and anti-pseudomonal β-lactams (such as ceftazidime, cefepime, or piperacillin-tazobactam) should be administered. When confronting challenges like carbapenem-resistant Enterobacterales, treatment should utilize novel β-lactamase inhibitor combinations (such as meropenem-vaborbactam or ceftazidime-avibactam) tailored to specific resistance mechanisms. It should be emphasized that for cases with controlled infection sources (such as catheter removal), a 6-10 day short-course therapy guided by antimicrobial susceptibility testing has been demonstrated to be an effective and safe strategy that can reduce secondary fungal infections (You et al., 2024).

Studies demonstrate that meropenem/vaborbactam exhibits bactericidal activity against both planktonic and biofilm-associated cells in CRKP-induced CRBSI (Sivori et al., 2025). For Acinetobacter bacteremia, monotherapy with broad-spectrum β-lactams or combination therapy with amikacin/polymyxin appears effective (Güneş et al., 2025).

For fungal CRBSI management, current evidence supports differential therapeutic strategies for specific patient populations. Infections caused by Candida tropicalis may present with persistently negative blood cultures, requiring advanced diagnostic techniques like metagenomic next-generation sequencing (mNGS) for early diagnosis, followed by appropriate antifungal therapy with voriconazole (Dai et al., 2021). Studies demonstrate that micafungin and anidulafungin exhibit superior clinical efficacy in obese patients (body mass index, BMI >30 kg/m²) significantly improving survival rates and treatment success. Furthermore, newer-generation antifungals including rezafungin and fosmanogepix show promising clinical performance in this population, offering novel options for refractory fungal infections.

Atypical pathogens present additional therapeutic challenges. CRBSI cases caused by rare microorganisms such as Microbacterium paraoxydans require tailored antibiotic approaches, typically involving combination therapy with cefoperazone/sulbactam and gentamicin/urokinase catheter lock solutions (Tang et al., 2022). In a parallel case involving a CRBSI outbreak caused by Ralstonia mannitolilytica, all isolates demonstrated resistance to meropenem and piperacillin/tazobactam but remained susceptible to quinolones. The definitive treatment regimen employed either ciprofloxacin monotherapy or its combination with cefepime. These cases underscore the critical importance of guided antimicrobial stewardship—particularly reliance on precise antimicrobial susceptibility testing profiles—when formulating individualized therapeutic strategies against uncommon pathogens (Fabricci et al., 2023).

This therapeutic strategy ensures clinical efficacy while minimizing unnecessary broad-spectrum antibiotic exposure, aligning with core principles of antimicrobial stewardship.

### 6.4 Catheter management strategies (retention, exchange, and removal)

Catheter management strategy constitutes a critical component of CRBSI management, with decision-making requiring comprehensive evaluation of infection severity, pathogen type, risk of complications, and patients’ long-term vascular access needs. A systematic approach to catheter salvage, which aims to clear the infection while preserving the vascular access, is a key consideration in selected cases. This strategy is centered on the combination of systemic antimicrobial therapy and adjunctive antimicrobial lock therapy (ALT).

Clear criteria should be met to justify an attempt at catheter salvage. Retention should only be considered if all the following conditions are fulfilled: (1) the infection is uncomplicated (hemodynamically stable, no evidence of metastatic infection or tunnel involvement); (2) the causative pathogen is not one typically mandating removal (e.g., Staphylococcus aureus, Pseudomonas aeruginosa, or fungi) and is susceptible to the planned lock therapy; (3) the patient demonstrates clear clinical improvement (e.g., defervescence, resolution of symptoms) within 48-72 hours of initiating appropriate therapy; and (4) bloodstream clearance is documented, ideally with follow-up blood cultures drawn from a peripheral vein (not via the catheter) showing no growth after 2-4 days of therapy. Indications for a catheter salvage attempt with ALT are typically restricted to uncomplicated CRBSI in hemodynamically stable patients, where the causative pathogen is highly susceptible to the chosen lock agent (often excluding *Staphylococcus aureus*, *Pseudomonas aeruginosa*, and fungi), and the patient has limited vascular access options.

A mandatory reassessment strategy is integral to the catheter salvage approach. Patients managed with an intent to retain the catheter must undergo a formal clinical and microbiological re-evaluation within 48-72 hours of initiating appropriate therapy. This reassessment should assess for clinical improvement (e.g., resolution of fever, stabilization of hemodynamics) and, crucially, for clearance of bacteremia through follow-up blood cultures. The presence of persistent bacteremia(positive blood cultures after 48-72 hours of effective therapy) is a strong indicator of failure of the salvage attempt and should prompt immediate re-evaluation, typically leading to the decision to remove the catheter.

When employed, ALT should use an agent (or combination) with documented efficacy against the identified pathogen and activity against biofilm.​ Common regimens include high-concentration solutions of antibiotics such as vancomycin (e.g., 2-5 mg/mL), gentamicin, or minocycline, often combined with an anticoagulant like heparin or citrate. Non-antibiotic lock solutions like citrate or ethanol are alternatives to mitigate antibiotic selection pressure. The duration of ALT typically parallels the course of systemic therapy, often ranging from 7 to 14 days, and should be guided by clinical and microbiological response.

In patients with Central Line-Associated Bloodstream Infection Present On Admission (CLABSI-POA), catheter removal can reduce mortality risk. For those with severe infections, sepsis/septic shock, or other catheter-related complications (e.g., septic thrombosis), immediate catheter removal is generally recommended. Catheter removal is typically required for Staphylococcus aureus, Pseudomonas aeruginosa, and multidrug-resistant organism infections (Hémar et al., 2025). Regarding fungal CRBSI management, current evidence supports tailored strategies for specific patient populations rather than universal catheter removal (Bond et al., 2023).

Under certain circumstances, catheter replacement may serve as a compromise strategy. Guidewire-assisted catheter exchange is appropriate for non-infectious catheter malfunctions such as intraluminal thrombosis or fibrin sheath formation, while creation of a new tunnel for catheter replacement should be considered when the original tunnel becomes infected or recurrent non-infectious catheter dysfunction occurs (Schröppel et al., 2025). For patients requiring vascular access despite active catheter infection, new catheter placement following effective antimicrobial therapy represents a viable option. A comparative study on Totally Implantable Venous Access Port (TIVAP)-related skin infections demonstrated that port salvage techniques offer minimally invasive and cost-effective alternatives to complete port reimplantation (Sun et al., 2025). No significant differences were observed in catheter infection-free survival between guidewire exchange and delayed catheter replacement after removal.

The timing of catheter reinsertion post-removal requires judicious determination. For bacterial CRBSI, patients still requiring CVC therapy need not delay reinsertion solely due to infection risk concerns. However, early reinsertion in candidal CRBSI may elevate persistent infection risks. Studies advocate determining CVC reinsertion timing based on actual patient needs rather than rigid adherence to temporal restrictions (Lee et al., 2021). Current guidelines generally recommend proceeding after bacteremia clearance and resolution of systemic infection symptoms to prevent new catheter reinfection. For high-risk patients, blood culture monitoring prior to new catheter placement may be considered to confirm complete infection eradication.

The management of HD-CRBSI requires a structured approach integrating rapid diagnosis, risk stratification, and decisive catheter management. A proposed clinical algorithm to guide decision-making, from initial suspicion through treatment evaluation, is presented in **Figure 2**. This integrated diagnostic and therapeutic flowchart (**Figure 2**) outlines key decision nodes. Initial assessment focuses on the patient’s hemodynamic stability; instability mandates immediate catheter removal. For stable patients, the identified pathogen becomes the next critical branch point: infections with Staphylococcus aureus, Pseudomonas aeruginosa, or fungi generally favor catheter removal, whereas those caused by other organisms may allow for retention strategies with concurrent antimicrobial therapy. Subsequent decisions further consider the presence of complications and the success of initial therapy, guiding choices between catheter retention, exchange over a guidewire, or delayed reinsertion.

### 6.5 Antimicrobial stewardship in HD-CRBSI management

Antimicrobial stewardship is a critical pillar in the management of HD-CRBSI, essential for optimizing clinical outcomes while mitigating the emergence and spread of antimicrobial resistance, a concern highlighted in preceding sections. Its implementation revolves around three core principles: timely de-escalation, therapeutic drug monitoring (TDM), and adherence to defined treatment durations.

De-escalation of therapy is fundamental. Once blood culture and susceptibility results are available, empirical broad-spectrum regimens should be rapidly streamlined to the most targeted, narrowest-spectrum agent effective against the identified pathogen. This practice, directly informed by the susceptibility-guided strategies discussed in Section 6.2, minimizes unnecessary antimicrobial exposure, a key driver of resistance.

TDM is indispensable for optimizing the efficacy and safety of key agents like vancomycin. Given its variable pharmacokinetics in hemodialysis patients, monitoring vancomycin trough concentrations (typically targeting 15-20 mg/L for serious MRSA infections) is mandatory to ensure therapeutic levels and avoid under-dosing (which promotes resistance) or over-dosing (which increases nephrotoxicity risk). TDM guides precise dose adjustments in the post-dialysis period.

Finally, adherence to evidence-based treatment durations is crucial. Overtreatment increases resistance pressure without added benefit, while under treatment risks relapse. Management must be guided by the specific pathogen, complication status, and whether the catheter is retained or removed, as outlined in Section 6.2. For example, uncomplicated bacteremia with catheter removal may permit a 7-day course for coagulase-negative staphylococci, whereas *S. aureus bacteremia* typically requires a minimum of 14 days. A structured AMS program that enforces these principles—de-escalation, TDM, and duration control—is integral to curbing the rising threat of multidrug-resistant organisms in the HD population.

## 7 Special considerations and future perspectives

### 7.1 Management of special populations

Prevention and management of CRBSI in special populations require individualized strategies. Within the chronic hemodialysis population, certain subgroups present distinct challenges and require tailored approaches.

Patients with Diabetes Mellitus or Malnutrition:​ Diabetes is a common comorbidity in HD patients and is associated with impaired wound healing, immune dysfunction, and an increased risk of infection. Special attention to glycemic control, meticulous exit-site care, and vigilance for signs of infection are crucial. Malnutrition, often indicated by hypoalbuminemia, is a marker of protein-energy wasting and systemic inflammation, which directly compromises immune function and increases susceptibility to and mortality from CRBSI. Nutritional support is an important adjunctive measure.

Patients with Obesity (particularly BMI ≥40):​ Severe obesity is an established independent risk factor for catheter-related infections. Management challenges include technical difficulties during catheter insertion and securement, potentially deeper catheter tunnels, and altered pharmacokinetics of antibiotics. Preventive measures should be adapted, such as ensuring adequate sterile draping, using appropriate securement devices, considering catheter length, and implementing rigorous TDM for antibiotics.

Patients Colonized or Infected with MDRO: Patients with a known history of MDRO (e.g., MRSA, VRE, carbapenem-resistant Gram-negative bacteria) colonization or prior infection represent a high-risk subgroup. This history should guide the choice of empiric antibiotic therapy and may influence decisions regarding the use of prophylactic or therapeutic antimicrobial lock solutions. Enhanced infection control precautions are also warranted.

Patients with Extremely Limited Vascular Access: For patients who have exhausted all options for arteriovenous fistulas or grafts and are entirely catheter-dependent, the decision between catheter salvage and removal becomes even more complex. In this scenario, a trial of catheter salvage with combined systemic and lock therapy may be more strongly considered for an uncomplicated CRBSI caused by a susceptible pathogen, even as the criteria for re-assessment and failure remain strict.

Geriatric patients face unique challenges in managing CRBSI, with hospitalization and overall 90-day mortality rates reaching 29%. For geriatric hemodialysis patients, an autologous arteriovenous fistula should be prioritized whenever possible. If TCVC are required, the following preventive measures must be reinforced: strict daily catheter disinfection, preferential coverage of drug-resistant bacteria, prompt removal of infected catheters, avoidance of inappropriate antibiotic use, and vigilance for hemodynamic instability (Dussartre et al., 2025).

Effective prevention and management in these HD-specific subgroups rely on early risk identification, strict adherence to augmented catheter care protocols, and individualized decision-making that weighs the risk of infection against the imperative of preserving vital vascular access.

### 7.2 Emerging technologies and research directions

#### 7.2.1 Technologies approaching clinical application

##### 7.2.1.1 Antimicrobial catheter coatings

Chitosan/copper composite coatings have demonstrated up to 99.9% bactericidal activity *in vitro* and in animal models, with sustained antimicrobial effects over prolonged immersion (Gu et al., 2022). These coatings are currently in late-stage preclinical evaluation. Similarly, heparin-coated catheters designed to reduce thrombus formation—a known nidus for bacterial colonization—are already in clinical use in some settings, though HD-specific efficacy data remain limited.

##### 7.2.1.2 Artificial intelligence-assisted surveillance

Machine learning models for early detection of vascular catheter-associated infections have shown promising performance in retrospective clinical datasets, with balanced accuracy exceeding 80% in some studies (Spaimoc et al., 2025). Automated surveillance algorithms incorporating site-specific microbiological culture results demonstrate high specificity and are being piloted in several healthcare systems (Januel et al., 2023). These tools require prospective multicenter validation before routine clinical deployment.

#### 7.2.2 Preclinical and experimental strategies

##### 7.2.2.1 Bacteriophage-enzyme composite coatings

Phage-based catheter coatings represent a novel biological approach to preventing multidrug-resistant bacterial colonization (Wójcicki et al., 2025). These bioagents demonstrate precise antibacterial targeting and the ability to disrupt established biofilm structures. However, all supporting data remain at the *in vitro* and animal model stage, and substantial development work is required before clinical translation.

##### 7.2.2.2 Vaccine development

Protein subunit vaccines targeting Acinetobacter baumannii adhesin Abp2D have demonstrated promising immunoprotective effects in murine catheter-associated infection models (Timm et al., 2023). While this strategy holds potential for durable immune protection in high-risk patients with prolonged catheter use, human trials have not yet commenced.

##### 7.2.2.3 Nitric oxide-releasing and other bioactive coatings

Experimental coatings incorporating nitric oxide donors or superhydrophilic antifouling ligands have demonstrated biofilm prevention in laboratory settings (Goodman et al., 2025). These remain at the preclinical proof-of-concept stage.

In summary, while several emerging technologies show promise for HD-CRBSI management, most remain in experimental phases. The near-term clinical impact is most likely to come from antimicrobial-coated catheters and AI-assisted surveillance tools, whereas phage-based strategies and vaccines represent longer-term horizons. Future research should prioritize prospective clinical validation of the most promising candidates (Frödin et al., 2025; Jin et al., 2025).

Furthermore, existing evidence faces limitations due to inconsistent CRBSI definitions and therapeutic evaluation criteria, necessitating international consensus to standardize study designs. Promoting unified infection definitions and standardized surveillance methods will also establish a robust foundation for comparing infection rates across institutions and evaluating intervention effectiveness (Pal et al., 2023).

## 8 Conclusions

HD-CRBSI remains a critical clinical challenge for patients with ESRD, characterized by high incidence, complex etiology, and multidrug resistance, all of which significantly impact patient outcomes and healthcare resource utilization. This narrative review synthesizes current evidence on the epidemiology, microbiological profiles, pathogenic mechanisms, and preventive and therapeutic strategies for HD-CRBSI. It underscores the necessity of implementing a multi-layered, integrated management framework. Key components include standardized catheter insertion and maintenance techniques, judicious use of antimicrobial-coated catheters, development of novel lock solutions, execution of evidence-based care bundles, and incorporation of intelligent diagnostic tools. Therapeutically, management should be guided by pathogen identification and individual patient factors, involving tailored antimicrobial regimens and reasoned decisions regarding catheter retention, exchange, or removal. Future research should prioritize establishing standardized surveillance systems, applying implementation science to infection control, and developing next-generation prevention and treatment strategies. Through multidisciplinary collaboration and translational efforts, these approaches can further reduce the burden of HD-CRBSI and enhance the long-term quality of life for hemodialysis patients.
